# EphB4/EphrinB2 therapeutics in Rhabdomyosarcoma

**DOI:** 10.1371/journal.pone.0183161

**Published:** 2017-08-17

**Authors:** Matthew E. Randolph, Megan M. Cleary, Zia Bajwa, Matthew N. Svalina, Michael C. Young, Atiya Mansoor, Pali Kaur, Carol J. Bult, Martin W. Goros, Joel E. Michalek, Sunny Xiang, James Keck, Valery Krasnoperov, Parkash Gill, Charles Keller

**Affiliations:** 1 Children’s Cancer Therapy Development Institute, Beaverton, Oregon, United States of America; 2 Department of Pediatrics, Oregon Health & Science University, Portland, Oregon, United States of America; 3 Department of Pathology, Oregon Health & Science University, Portland, Oregon, United States of America; 4 The Jackson Laboratory Cancer Center, The Jackson Laboratory, Bar Harbor, Maine, United States of America; 5 Department of Epidemiology and Biostatistics, University of Texas Health Science Center, San Antonio, Texas, United States of America; 6 Vasgene Therapeutics, Los Angeles, California, United States of America; Cedars-Sinai Medical Center, UNITED STATES

## Abstract

Rhabdomyosarcoma (RMS) is the most common soft tissue sarcoma affecting children and is often diagnosed with concurrent metastases. Unfortunately, few effective therapies have been discovered that improve the long-term survival rate for children with metastatic disease. Here we determined effectiveness of targeting the receptor tyrosine kinase, EphB4, in both alveolar and embryonal RMS either directly through the inhibitory antibody, VasG3, or indirectly by blocking both forward and reverse signaling of EphB4 binding to EphrinB2, cognate ligand of EphB4. Clinically, EphB4 expression in eRMS was correlated with longer survival. Experimentally, inhibition of EphB4 with VasG3 in both aRMS and eRMS orthotopic xenograft and allograft models failed to alter tumor progression. Inhibition of EphB4 forward signaling using soluble EphB4 protein fused with murine serum albumin failed to affect eRMS model tumor progression, but did moderately slow progression in murine aRMS. We conclude that inhibition of EphB4 signaling with these agents is not a viable monotherapy for rhabdomyosarcoma.

## Introduction

Rhabdomyosarcoma (RMS) is a highly aggressive tumor with myogenic features and ranks as the most common soft tissue cancer in children. RMS poses significant challenges to treatment because more than half of tumors metastasize early in the disease [1]. Metastatic forms of the two most common subtypes of RMS, alveolar and embryonal, are associated with especially poor clinical outcomes [1, 2]. Long-term survival rates of children with rhabdomyosarcoma have not improved in over thirty years, with survival rates of 40% for embryonal rhabdomyosarcoma (eRMS) and less than 20% for alveolar rhabdomyosarcoma (aRMS) [2, 3]. New therapeutic targets that address tumor invasion and metastasis are urgently needed to improve patient outcomes.

The process of invasion and metastasis in the context of the tumor microenvironment of RMS is similar to embryonic tissue patterning, and receptor tyrosine kinases (RTKs) play critical roles in both of these processes [4]. The Eph proteins are the largest class of receptor tyrosine kinases and they have emerged as major determinants in both cancer and normal tissue development [5]. Eph proteins and their ephrin ligands participate in complex bidirectional signaling pathways that promote epithelial phenotypes and regulate cell adhesion, migration, and vascular remodeling [5]. Although the role of Eph proteins in rhabdomyosarcoma has been largely uncharacterized until recently, earlier reports have affirmed upregulation of Ephrin receptor B4 (EphB4) and its cognate ligand EphrinB2 in aRMS [6–8]. We have since identified EphB4 as a pharmacological target and potential two-way switch in the context of aRMS [9]. Here, we explore the effects of inhibiting EphB4/EphrinB2 signaling in preclinical RMS models via two independent small-molecule inhibitors: the investigational VasG3 (anti-EphB4) antibody [10], and an EphrinB2 decoy receptor, the soluble EphB4-HSA (human serum albumin) fusion protein or the related soluble EphB4-MSA (mouse serum albumin) fusion protein.

## Methods and materials

### Human samples

Tissue microarrays (TMAs) of human embryonal rhabdomyosarcomas (#3000-30-P8968); neuroblastomas (#3000-30-P7338); and osteosarcomas (#3000-30-P9339) were provided by the Children’s Oncology Group Biorepository (Columbus, OH). Additionally, we constructed publicly-available TMAs of both murine and human rhabdomyosarcoma tumors in collaboration with the BioPathology Center (Nationwide Children’s Hospital, Columbus, OH). All TMAs were constructed from formalin-fixed tissue and provided as unstained sections, 4 microns thick, mounted on charged glass slides. Human osteosarcoma and neuroblastoma primary cultures were generated from tumor fragments obtained from surgical samples. Immunohistochemistry and immunoblotting studies were performed under an Institutional Review Board-approved protocol (cc-TDI).

### Cell lines and cultures

The murine aRMS cell line, U48484, was derived from a *Myf6*^*ICNm/WT*^ *Pax3*^*P3Fm/P3Fm*^ *Trp53*^*F2-10/F2-10*^ transgenic mouse, previously described [9]. Human cell line and xenograft models are described in Table 1. The commercially available human cell lines U-2 OS and SH-SY5Y, purchased from the American Type Culture Collection (Manassas, VA), were utilized to assess osteosarcoma and neuroblastoma sensitivities to VasG3 treatment, respectively. Additionally, the human alveolar rhabdomyosarcoma (aRMS) cell line, Rh5, was generously provided by the cell line originator, Peter Houghton of St. Jude Children’s Research Hospital, Memphis, TN. The primary osteosarcoma culture, PCB151, was derived from surgical biopsy of an aggressive osteosarcoma of the distal femur of an 11-year-old male. Cells were grown with either RPMI 1640 (Life Technologies, St. Louis, MO) for human cells or Dulbecco’s Modified Eagle’s Medium (DMEM) (Life Technologies) for murine cells, respectively supplemented with 10% fetal bovine serum (FBS) (Atlanta Biologicals, Atlanta, GA) and penicillin (100 U/mL)/streptomycin (100 μg/mL) (Thermo Fisher Scientific, Waltham, MA) in 5% CO_2_ at 37°C.

**Table 1:** Features of human xenograft model systems.

Model	Source	Age	Gender	Histology	Site	Metastasis	Reference
PCB82	Autopsy	14 y	Female	eRMS	Leptomeningeal	No	[11]
PCB380	Surgical	2 y	Female	aRMS	Limb	————	This report
Rh18	————	2 y	Female	eRMS	Perineum	————	[9, 12, 13]
Rh30	————	16 y	Male	aRMS	Bone marrow met	Yes	[9, 13, 14]

### Immunoblotting

Cells or tumor fragments were lysed with Pierce RIPA Buffer (Thermo Scientific) containing 1:100 dilutions of Halt Protease and Phosphatase Inhibitor Cocktail 100X (Thermo Scientific) on ice, centrifuged and supernatant collected. Protein concentration of the supernatant was determined by bicinchoninic acid (BCA) assay using Pierce BCA Protein Assay Kit (Thermo Scientific). Standard immunoblotting procedures were then performed on equivalent amounts of protein per sample. Briefly, 20–30 μg of protein samples were separated via sodium dodecyl sulfate polyacrylamide gel electrophoresis (SDS-PAGE) using 4–20% Mini-PROTEAN TGX gels (Bio-Rad Laboratories, Hercules, CA) and subsequently transferred to Immun-Blot® PVDF (polyvinylidene difluoride) membranes (Bio-Rad Laboratories). Blots were blocked with 5% bovine serum albumin (BSA; Santa Cruz Biotechnology, Santa Cruz, CA) in a mixture of Tris-buffered saline and Tween-20 (TBS-T, 0.2% Tween) and incubated with anti-EphB4 (265, mouse IgG1; 2 μg/ml; Vasgene Therapeutics, Los Angeles, CA), anti-EphrinB2 (2B5, mouse IgG1; 4 μg/ml; Vasgene Therapeutics), anti-Src (sc-19, rabbit polyclonal IgG; 0.2 μg/ml; Santa Cruz Biotechnology), anti-phospho-Src (sc-81521, mouse monoclonal IgG; 0.4 μg/ml; Santa Cruz Biotechnology), anti-α-tubulin (ab4074, rabbit polyclonal IgG; 1μg/ml Abcam, Cambridge, MA) or anti-β-actin (ab8227, rabbit polyclonal IgG; 50 ng/ml; Abcam) in blocking solution overnight at 4°C. Blots were subsequently incubated with species appropriate secondary antibodies conjugated to horseradish peroxidase (HRP; 0.1 μg/ml; Vector Laboratories Inc., Burlingame, CA) in blocking solution for 1 hour at room temperature (RT). The resulting immune complexes were visualized via chemiluminescence using Clarity Western ECL Substrate (Bio-Rad Laboratories) and densitometry analysis performed using ImageJ Version 1.43u [15].

### Immunohistochemistry

Histology was performed as follows: formalin-fixed paraffin embedded sections were treated with xylene and rehydrated via graded alcohol concentrations to remove paraffin. Heat-induced epitope retrieval was performed with 10 mM sodium citrate buffer (pH 6.0, 0.05% Tween) for 20 minutes. Endogenous peroxidases were quenched using 3% hydrogen peroxide in phosphate buffered saline (PBS) for 10 minutes at RT. Slides were blocked in 1% horse serum in PBS in the dark for 1 hour at RT. Primary antibodies for EphB4 (sc-5536, rabbit polyclonal; Santa Cruz Biotechnology) and EphrinB2 (sc-1010, rabbit polyclonal; Santa Cruz Biotechnology) were used at 0.8 μg IgG/ml in blocking solution overnight at 4°C. Slides were then washed in PBS and incubated in secondary antibody (biotinylated goat anti-mouse anti-rabbit IgG; Vector Laboratories) at a 1:400 dilution in blocking solution in the dark for 1 hour at RT. The VECTASTAIN Elite ABC kit (Vector Laboratories) and ImmPACT™ DAB Peroxidase Substrate kit (Vector Laboratories) were used per manufacturer protocols to visualize staining. Slides were counterstained with hematoxylin (Vector Laboratories) for 5 minutes, rinsed with tap water, dehydrated via graded alcohol concentrations, and mounted with Richard-Allan Scientific Cytoseal XYL mounting medium (Thermo Scientific). A board-certified pathologist (AM) then analyzed and graded slides for staining intensity. Images were obtained using a Nikon Coolscope and Coolscope VS software (Nikon, Melville, NY).

### *In vitro* cell viability assays

Cell viability assays utilizing the CellTiter-Glo® Luminescent Cell Viability Assay (Promega, Madison, WI) were performed using serial dilutions of 0.05 nM-1μM h131 [10] (VasG3, Vasgene Therapeutics) on alveolar rhabdomyosarcoma, osteosarcoma, and neuroblastoma primary cultures and cell lines, described above. Briefly, cells were cultured as described above. Adherent cells were washed with sterile phosphate buffered saline (11.9 mM phosphates, 137 mM sodium chloride and 2.7 mM potassium chloride, PBS), and detached by incubating in 0.05% Trypsin/EDTA (Life Technologies) at 37°C for 2–3 minutes. Cells were then transferred to 96-well plates with 5000 cells/well. Serial dilutions of VasG3 IgG or midostaurin (Selleckchem.com, Houston, Texas) were then added in quadruplicate, using parallel serial dilutions of isotype IgGs (Innovative Research, Novi, MI) or dimethyl sulfoxide (Sigma-Aldrich, Saint Louis, MO) as negative controls, respectively. Cells were then incubated at 37°C, in 5% CO2, for 72 hours prior to addition of the CellTiter-Glo® luminescent substrate. Luminescence was quantified using the *Synergy HT* plate reader (BioTek Instruments, Winooski, VT) with Gene5 2.03 software (BioTek Instruments). IC50s and IC25s were calculated for each cell line/culture using Prism 6 for Mac OS X, Version 6.0f (GraphPad Software, La Jolla, CA). Experiments were performed in quadruplicates for each cell line/culture.

### *In vivo* therapeutic studies

*In vivo* xenograft and allograft experiments were performed using five- to six-week-old female *NOD*.*Cg-Prkdc*^*scid*^ *Il2rg*^*tm1Wjl*^*/SzJ* (NSG) mice (Jackson Laboratory). Mice were randomly divided into cohorts of 12 mice per group and 3–5 mm^3^ rhabdomyosarcoma fragments (passage 1–3) were trocar implanted in the right hind flank. Experimental mice were subjected to one of two studies. 1) Mice were injected intravenously every seven days with 10 mg/kg h131 [10] (Vasgene Therapeutics) for the human xenograft studies. Control cohorts received intravenous injections of human isotype control IgG, Protein A purified (Innovative Research, Novi, MI). The control cohorts were treated every 7 days for a total of 6 treatments in parallel with the experimental cohorts. 2) Mice were injected intraperitoneally three times a week with 50 mg/kg of soluble EphB4 conjugated to either human or mouse serum albumin (respectively sEphB4-HSA or sEphB4-MSA). Control cohorts were injected three times a week intraperitoneally with 50 mg/kg HSA or MSA for human xenografts and mouse allografts, respectively. For both groups of experimental studies, body weight and tumor volume were recorded twice weekly once tumors reached a measurable size of 200–300 mm^3^. Mice were monitored until the experimental endpoint of 60 days post-trocarization or a tumor volume equaling or exceeding 2000 mm^3^. Mice were euthanized by CO_2_ asphyxiation once a study endpoint was reached. Upon euthanasia, lungs and tumors were collected. The left lungs were snap-frozen in liquid nitrogen and stored at -80°C for biochemical processing while the right lungs were fixed in 10% buffered formalin for histologic analysis. Tumors were cut into halves with one half snap-frozen, as described above, and the other half fixed in 10% buffered formalin. Experiments were performed in accordance with approved guidelines and ethical approval from both Oregon Health and Science University and Jackson Laboratory Institutional Animal Care and Use Committees and in compliance with the National Institutes of Health.

### Statistical analysis

When two sets of data were compared, student *t* tests were performed to determine statistical significance, establishing the probability of significance at less than or equal to 0.05. The Gehan-Breslow-Wilcoxon analysis of survival curves was applied to determine significance of *in vivo* experiments using Prism 6 for Mac OS X, Version 6.0f (GraphPad Software, La Jolla, CA). Statistical data for all experiments was reviewed and confirmed by JM.

## Results

### *In vivo* inhibition of EphB4 in aRMS using the inhibitory VasG3 antibody does not affect tumor growth

**Fig 1:**
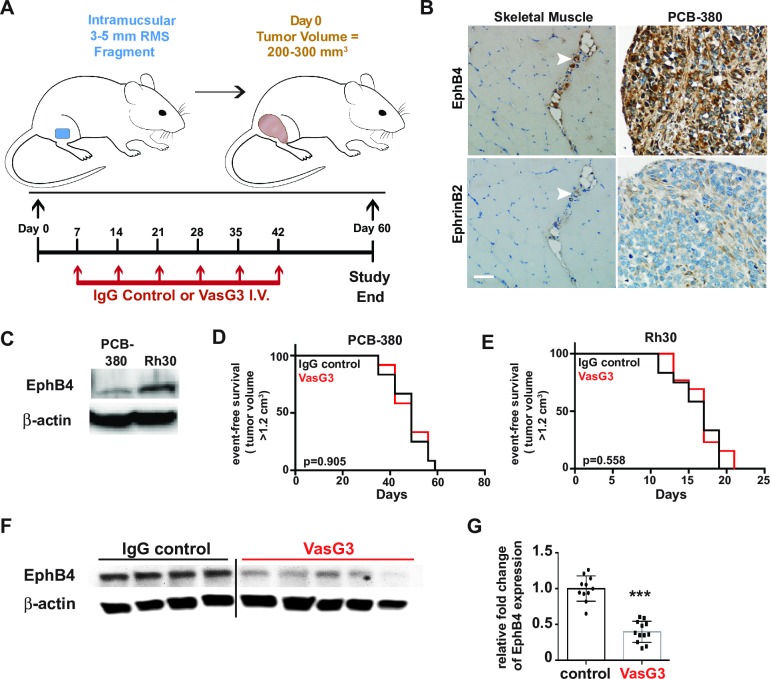
VasG3 treatment of Rh30 & PCB380 aRMS xenografts. *A*. Experimental design of the orthotopic aRMS xenograft tumor models and VasG3 treatment regime. *B*. Immunohistochemical staining of the patient-derived PCB380 tumor to demonstrate EphB4 and EphrinB2 expression as obtained from the BioPathology Center RMS tissue microarray. Skeletal muscles sections were concurrently stained as controls. Arrow heads indicate EphB4/EphrinB2 expressing vascular/stromal cells. Mag bar = 50 μm. *C*. EphB4 protein expression in cultured aRMS cell lines. *D and E*. Kaplan-Meier survival curves post-treatment initiation of isotype control (black line) versus VasG3 (anti-human EphB4 binding antibody; red line) treated mice for both the PCB380 (*D*) and Rh30 (*E*) xenograft models. Mice were euthanized either on day 60 or when the tumor volume exceeded 1.2 cm^3^. n = 11–12 female mice per treatment group for both models. *F and G*. Representative western blot demonstrating loss of EphB4 protein levels with VasG3 treatment (*F*) and quantification of EphB4 loss (*G*) for the Rh30 model. n = 11–12 mice per cohort. p<0.0001.

Previously, we have demonstrated that EphB4 and EphrinB2 are both expressed in the alveolar subtype of rhabdomyosarcoma (aRMS) [9]. EphB4 expression was associated with decreased survival times compared to patients with tumors of little to no EphB4 expression [9]. Importantly, EphB4 receptor tyrosine kinase signaling has been associated with oncogenic Pax3:Foxo1 fusion protein expression and involved in aRMS tumor progression [9]. Therefore, to determine whether EphB4 signaling was a viable therapeutic target for clinical trials, we first analyzed EphB4 inhibition in aRMS since EphB4 expression is increased in this RMS subtype relative to striated muscle [9]. We tested two different orthotopic xenograft models of human aRMS (Fig 1A) in 5 to 6-week old NOD scid gamma (NSG) mice. PCB380 is a patient-derived primary aRMS tumor while Rh30 is a human aRMS cell line, both of which express both EphB4 and EphrinB2 proteins [9] (Fig 1B and 1C). Hind limb muscles were injured via cardiotoxin injection 24 hours prior to injection of Rh30 cells. In contrast, primary tumor fragments of PCB380 were trocar implanted into the muscles of the hind flank. Mice were then randomly divided into two cohorts of 12 mice and subjected to intravenous injections of either isotype IgG control antibody or VasG3 (anti-EphB4 Ig [10]) antibody once weekly for either six weeks or until tumors reached a size of 2000 mm^3^ for both aRMS models. Treatment with the humanized EphB4 inhibitory antibody failed to affect tumor growth or survival rates in both the PCB380 and Rh30 xenograft models when compared to the IgG control group (Fig 1D and 1E). EphB4 has previously been shown to be internalized and degraded through endocytosis when inhibited [10]; therefore, pharmacodynamics of EphB4 expression were assessed via western blot. Loss of EphB4 protein levels occurred with VasG3 antibody treatment in the Rh30 xenograft model (Fig 1F and 1G). However, EphB4 levels were too low to detect any pharmacodynamic change via western blot in the PCB380 patient-derived xenograft (PDX) mice (data not shown). In addition, *in vitro* treatment with serial dilutions of VasG3, ranging from 0.05 nM to 1 μM, failed to induce cell death (S1A Fig). To rule out the possibility of VasG3 having a synergistic effect with other kinase inhibitors, Rh30 aRMS cells were treated with serial dilutions of the pan-kinase inhibitor, midostaurin (PKC412), in the presence of 10 nM VasG3. No differences in IC_25_ concentrations were observed following the addition of VasG3 (S1B Fig). These data indicate that single agent inhibition of EphB4 with an inhibitory antibody is not sufficient to adversely affect aRMS growth or survival *in vivo*, and that the effect with a pan-kinase inhibitor was not synergistic.

### *In vivo* inhibition of EphB4/EphrinB2 forward signaling pathways in aRMS model systems

**Fig 2:**
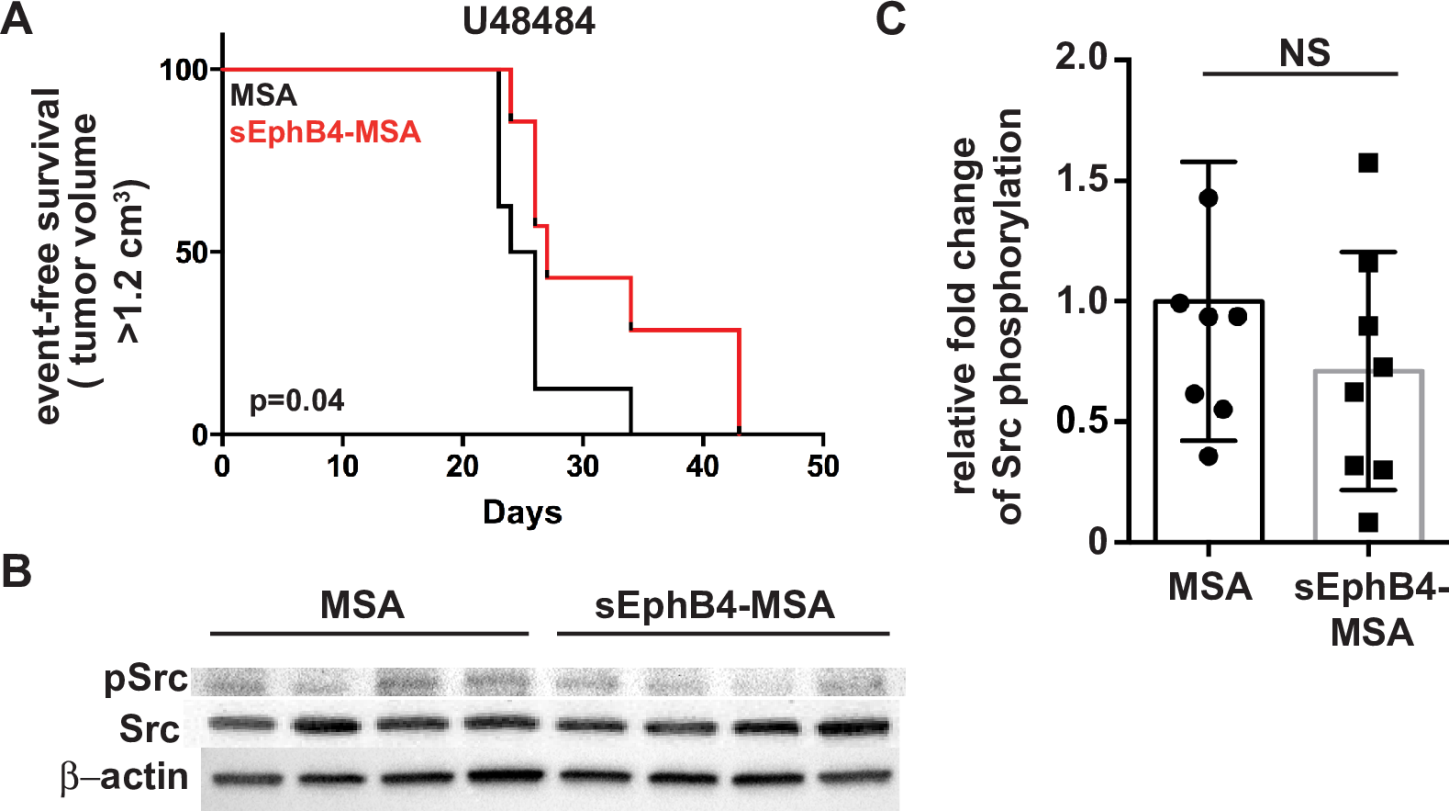
Effect of blocking EphrinB2 signaling by sEphB4-MSA in murine aRMS allograft models. *A*. Kaplan-Meier curve for treatment with sEphB4-MSA, a sump that binds and sequesters EphrinB2; n = 11–12 female mice per cohort. Black line, mouse serum albumin (MSA) treatment. Red line, soluble EphB4-MSA treatment. *B*, *C*. Western blots demonstrating the pharmacodynamic effect of soluble EphB4-MSA treatment on Src phosphorylation of tyrosine 418 in U48484 allograft tumors (*B*). No significant differences were observed with densitometry quantitation (*C*). n = 7–8 mice per cohort. p = 0.3001.

EphB4 signaling can be induced by *trans* interactions with its cognate ligand, EphrinB2, typically expressed in the tumor microenvironment on endothelial [5]; or by *cis* interactions with PDGFRβ expressed on RMS tumor cells [9]. EphB4 forward signaling with EphrinB2 has been associated with an increase in aRMS apoptosis [9]. However, when EphrinB2 protein expression is impaired via siRNA, aRMS cell viability is minimally affected [9]. Therefore, to assess the role of EphB4/EphrinB2 signaling on aRMS tumor progression, we competitively inhibited EphB4 forward signaling using a soluble EphB4 linked either to mouse or human serum albumin (sEphB4-MSA, sEphB4-HSA) [16] in both orthotopic allograft and xenograft models using 5 to 6-week old NOD scid gamma (NSG) mice. We initially tested the *in vivo* effects of sEphB4-MSA verses MSA treatment of mice injected with U48484, a murine aRMS cell culture derived from a *Myf6*^*ICNm/WT*^ *Pax3*^*P3Fm/P3Fm*^ *Trp53*^*F2-10/F2-10*^ transgenic mouse [9]. Hind limb muscles were injured via cardiotoxin injection 24 hours prior to injection of U48484 cells into the muscles of the hind flank. Mice were then randomly divided into two cohorts of 8 mice and subjected to intraperitoneal injections of either MSA control or sEphB4-MSA three times weekly for six weeks. EphrinB2 reverse signaling, through the Src pathway, was not affected as demonstrated by absence of differential Src phosphorylation at tyrosine 418 (Fig 2B and 2C), the site of Src autophosphorylation induced through EphB4/EphrinB2 reverse signaling [17–19]. However, inhibition of EphB4/EphrinB2 forward signaling with sEphB4-MSA resulted in a decrease in U48484 tumor growth (Fig 2A; p = 0.043), suggesting that EphB4/EphrinB2 forward signaling contributes to aRMS progression *in vivo*.

### EphB4 expression is rare in human eRMS while also a positive prognostic indicator

**Fig 3:**
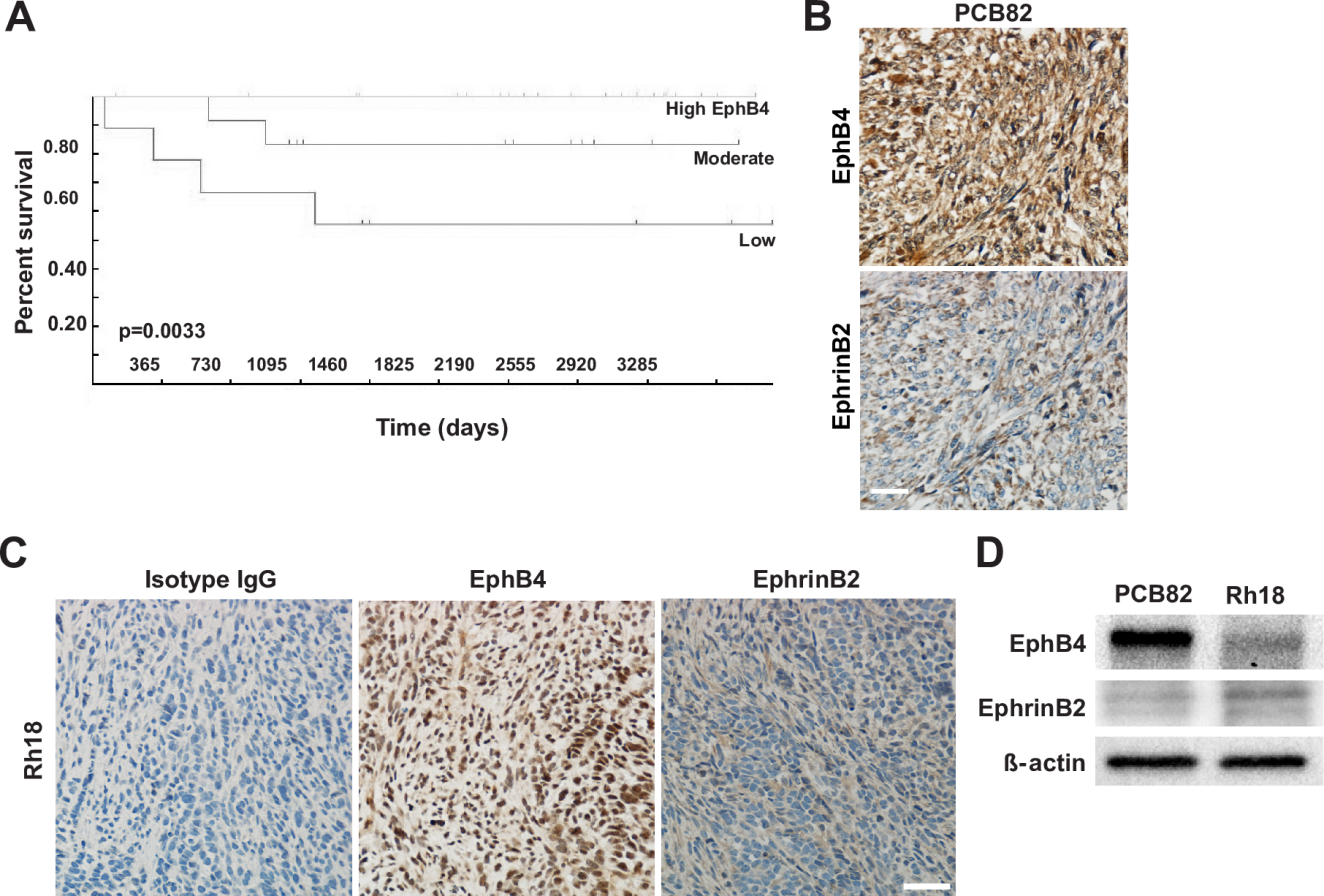
EphB4 and EphrinB2 expression in model systems of human eRMS. *A*. Patient survival improves with high EphB4 expression determined using the. p = 0.0033, n = 72. *B*. Human eRMS expression of EphB4 and EphrinB2, respectively, of PCB82 tissue sections from a human eRMS tissue microarray. Control skeletal muscle from the same TMA is depicted in Fig 1B. *C*. Human eRMS expression of EphB4 and EphrinB2 in Rh18 xenograft sections. Mag bar = 50 μm. *D*. Representative western blot demonstrating EphB4 and EphrinB2 protein expression in human eRMS xenograft models, PCB82 and Rh18.

As previously discussed, EphB4 and EphrinB2 are expressed in both human and murine aRMS; however, EphB4 and EphrinB2 expression in embryonal RMS (eRMS) has not been examined. Therefore, we queried the Intergroup Rhabdomyosarcoma Study Group (IRSG)-IV Affymetrix U95 GeneChip database [20] for expression of EphB4 in human eRMS and uncovered that elevated EphB4 expression correlates with a positive prognosis for eRMS patients (Fig 3A). We then tested the protein expression of EphB4 and its cognate ligand EphrinB2 in eRMS. Human eRMS tissue microarrays (TMAs) consisting of 38 unique cases were immunostained for the presence of EphB4 and EphrinB2 (Fig 3B). Expression levels were scored on a scale of 0–3 based on staining intensity. EphB4 was expressed in only 5% of eRMS, while over 39% of human eRMS expressed EphrinB2 (Table 2). Taken together, these data suggest a paradoxical role for EphB4 between the alveolar and embryonal human RMS subtypes, indicating that subtype-specific approaches for therapeutic intervention targeting EphB4/EphrinB2 signaling may be indicated. From the point of view of tool model systems, when murine soft tissue sarcoma TMAs were assessed as described above, EphB4 and EphrinB2 expression was observed in 86% and 57% of seven eRMS cases tested (Table 2).

**Table 2:** Immunohistochemistry of embryonal rhabdomyosarcoma tissue microarrays.

	Human				Murine			
	EphB4		EphrinB2		EphB4		EphrinB2	
	# of Samples	% of eRMS	# of Samples	% of eRMS	# of Samples	% of eRMS	# of Samples	% of eRMS
Stain present	2	7.4%	19	76%	9	100%	6	67%
% Positivity:								
0	25	92.5%	6	24%	0	0%	3	33%
1	1	3.7%	0	0%	0	0%	0	0%
2	1	3.7%	0	0%	1	11%	0	0%
3	0	0%	0	0%	2	22%	4	44%
4	0	0%	19	76%	6	67%	2	22%
Stain intensity:								
0	25	92.5%	6	24%	0	0%	3	33%
1	1	3.7%	6	24%	0	0%	2	22%
2	1	3.7%	10	40%	1	11%	3	33%
3	0	0%	3	12%	8	89%	1	11%
Fat	N/A	N/A	1	4%	1	11%	2	22%
Blood vessels	1	3.7%	8	32%	N/A	N/A	N/A	N/A
Stroma	N/A	N/A	3	12%	N/A	N/A	N/A	N/A

Human and murine eRMS TMAs were stained for EphB4 and EphrinB2 expression and assessed for staining intensities. N/A = not assessed.

### *In vivo* inhibition of EphB4/EphrinB2 forward signaling pathways in eRMS model systems

**Fig 4:**
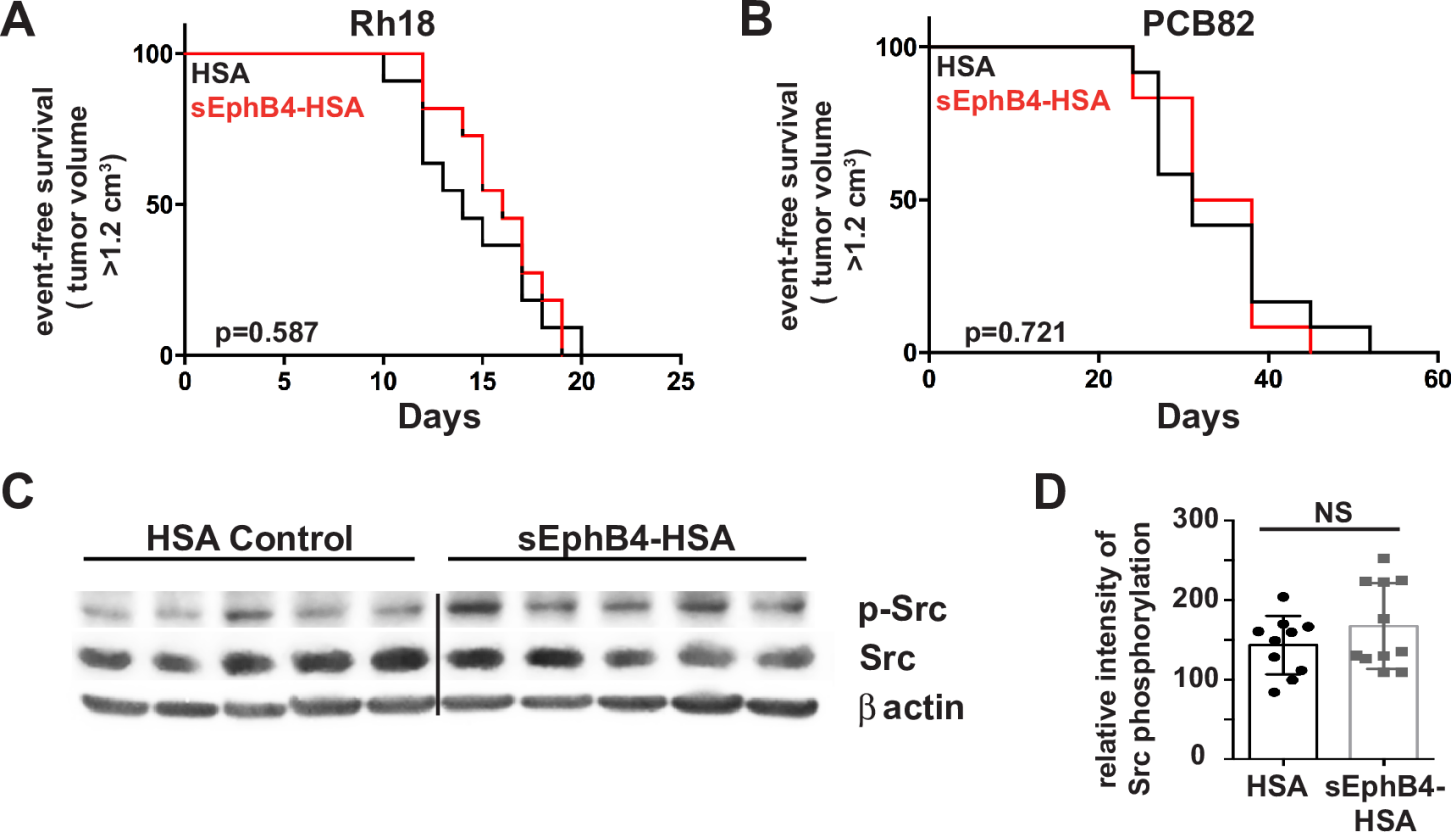
EphrinB2 blockade by sEphB4-HSA in human eRMS xenograft models. *A*, *B*. Kaplan-Meier curves for Rh18 (A) or PCB82 (B) xenograft models. n = 10–12 female mice per cohort per experiment. Black line, human serum albumin (HSA) control. Red line, soluble EphB4-HSA treatment. *C*, *D*. Western blots (C) showing Src phosphorylation from PCB82 human eRMS xenograft, lysates treated *in vivo* with either HSA or sEphB4-HSA were quantified for differences using densitometry (D). n = 10–11 female mcie per PCB82 cohort. p = 0.2539.

Considering the positive correlation between EphB4 expression and patient survival, we wanted to determine if EphB4 forward signaling inhibition was therapeutically contraindicated in the embryonal rhabdomyosarcoma subtype. Therefore, we developed and utilized two *in vivo* eRMS models using NSG mice: 1) an orthotopic patient-derived xenograft model using PCB82 [11], a patient-derived primary eRMS tumor; and 2) an orthotopic xenograft model using the eRMS cell line Rh18 [9, 12, 13]. We established the eRMS xenograft model by injecting mice with human Rh18 cells in hind limb muscle 24 hours post-cardiotoxin injury. The eRMS PDX model mice received primary tumor chunks of PCB82 via trocar implantation into the hind flank muscles. Both xenograft models demonstrated EphB4 and EphrinB2 protein expression as evidenced by immunohistochemistry and western blot (Fig 3C, 3D and 3E). Mice from both models received intraperitoneal injections of either human serum albumin (HSA) or sEphB4-HAS three times weekly for 6 weeks. Inhibition of EphB4/EphrinB2 forward signaling failed to affect eRMS tumor progression in either xenograft model (Fig 4A and 4B). Reverse signaling of EphrinB2 was not affected as evidenced by lack of changes in Src phosphorylation via western blot (Fig 4C and 4D). These data suggest that overall inhibition of EphB4 forward signaling is unlikely to provide therapeutic value for embryonal RMS patients.

### *In vivo* inhibition of EphB4 in eRMS using the inhibitory VasG3 antibody does not affect tumor growth

**Fig 5:**
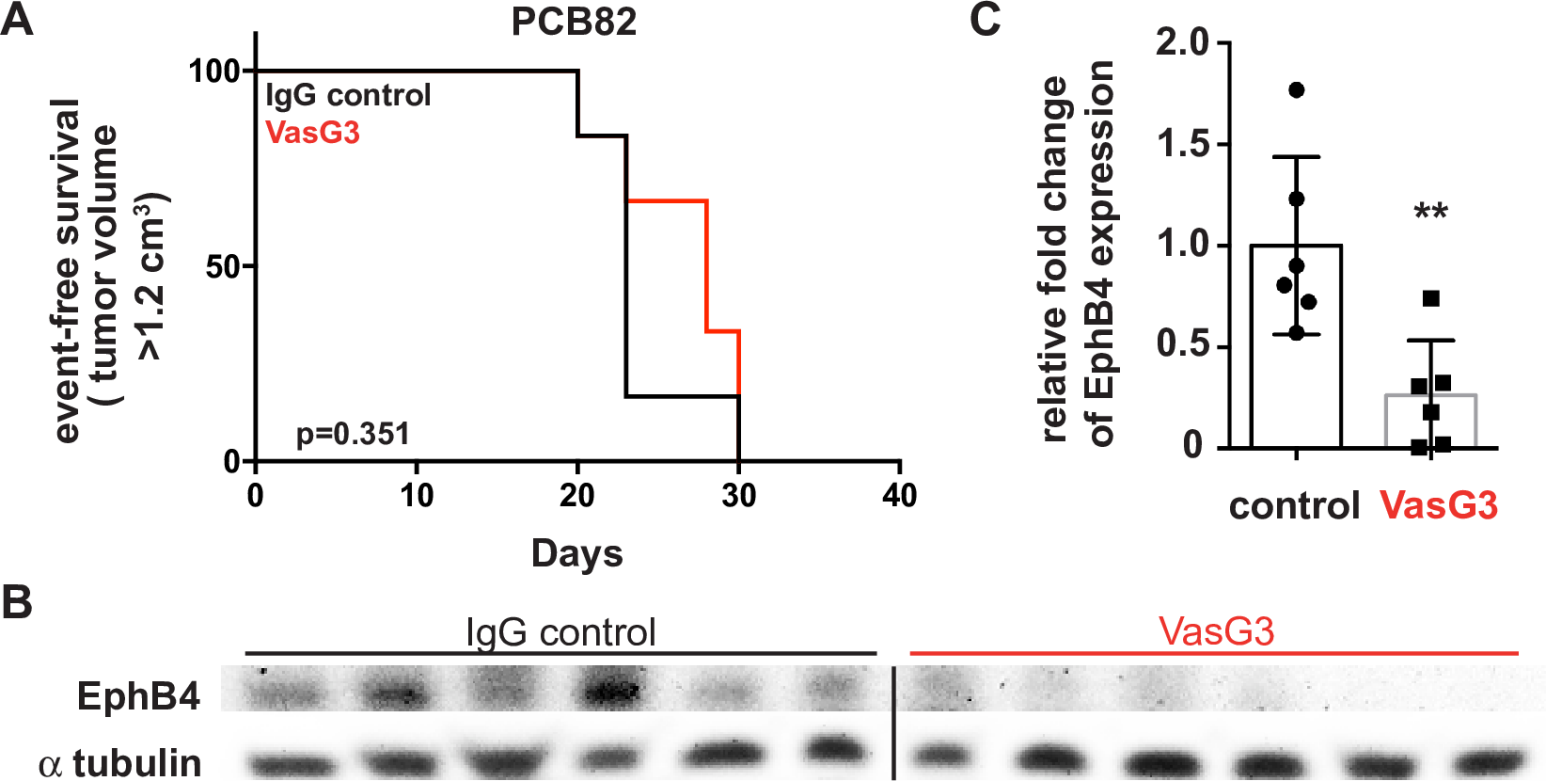
VasG3 treatment of PCB82 eRMS xenografts. *A*. Kaplan-Meier curve demonstrating percent event-free survival based on day post-treatment. Isotype control (black line) versus VasG3 (red line). p = 0.351, n = 6 female mice per cohort. *B*. Western blot demonstrating loss of EphB4 protein levels following VasG3 treatment. *C*. Differences in EphB4 protein levels following VasG3 treatment were quantified with densitometry. **p = 0.006.

To address the *in vivo* role of EphB4 inhibition in eRMS tumor progression, we utilized the EphB4 inhibitory antibody, VasG3, in an eRMS patient-derived xenograft (PDX) model. PCB82 xenograft mice were treated with VasG3 intravenously, as described in Fig 1, to test the effects of EphB4 inhibition on eRMS progression *in vivo*. VasG3 inhibition of EphB4 failed to decrease eRMS tumor growth when compared to the IgG control group (Fig 5A, p = 0.351). Pharmacodynamic loss of EphB4 protein was observed in the VasG3 treated cohort (Fig 5B and 5C, p = 0.006). These data suggest that EphB4 inhibition has no effect in extending survival time of eRMS patients whose tumors express EphB4.

### EphB4 and EphrinB2 expression in neuroblastoma and osteosarcoma cell lines

Considering the differential expression of EphB4 and EphrinB2 between the aRMS and eRMS, we decided to investigate whether EphB4 and EphrinB2 were expressed in other pediatric solid tumors, particularly neuroblastoma and osteosarcoma. We performed immunohistochemical assays for EphB4 and EphrinB2 expression on neuroblastoma tissue microarrays (TMAs) as well as osteosarcoma TMAs, which were then scored by a sarcoma pathologist (AM). For both receptors, moderate to high expression was prevalent in neuroblastoma (75%) and even more prevalent in osteosarcoma (83%) (S2A, S2B, S2C and S2D Fig). Additionally, using a survey set of patient-derived neuroblastoma samples and osteosarcoma samples, we observed an overall strong incidence of EphB4 and EphrinB2 expression in both tumor types (S2E Fig). However, representative cell lines for osteosarcoma (U-2 OS) and neuroblastoma (SH-SY5Y) did not respond to monotherapy with VasG3 (S1C and S1D Fig).

## Discussion

The development of novel therapeutics for metastatic pediatric rhabdomyosarcoma is critical for improving the long-term survival of affected patients. EphB4 has been associated with tumor growth, tumor suppression, and apoptosis in multiple types of cancers [21–26]. Here we utilized several *in vivo* mouse models of both alveolar and embryonal RMS to test the effectiveness of solely targeting the EphB4/EphrinB2 pathway as a potential therapeutic for childhood RMS. Our data demonstrate that inhibition of EphB4 using the EphB4 inhibitory antibody, VasG3, does not alter aRMS progression as a single therapeutic. However, inhibition of EphB4 forward signaling using soluble EphB4 resulted in slight decreases in murine aRMS tumor growth. These data did not correlate with an alteration in Src signaling, suggesting a possible off-target effect in murine aRMS, as we know EphB4 can interact with other cell signaling molecules [9]. Additional PDX studies examining sEphB4-HSA in human aRMS could, in the future, be performed to validate the positive effect on survival with EphrinB2 inhibition observed in the murine aRMS allograft study. Neither EphB4/EphrinB2 inhibition with soluble EphB4 nor VasG3 treatment demonstrated an effect on survival or tumor growth rates in human eRMS PDX models. Considering the negative results for VasG3 and sEphB4-HSA treatments in the human eRMS xenograft and PDX preclinical models, we believe these therapeutic agents should not be pursued as monotherapies for eRMS clinical trials.

Previously, we demonstrated that inhibition of EphB4 signaling in a murine aRMS allograft model using dasatinib, a receptor tyrosine kinase and Src kinase inhibitor, effectively decreased tumor progression [9]. The stark contrast between our current studies and the dasatinib study is likely because of targeting a single molecular pathway, EphB4/EphrinB2, verses multiple kinase targets and pathways. More information is needed to determine if combination therapies, in conjunction with EphB4 inhibition, could enhance the mild effects of EphB4/EphrinB2 inhibition in aRMS tumor progression. Finally, our expression data for EphB4 and EphrinB2 may indicate that further study of the EphB4 and EphrinB2 receptors in neuroblastoma and osteosarcoma could be warranted.

## Supporting information

S1 Fig *In vitro* effects of VasG3 treatment on various sarcomas as a single agent therapy. Cell viability of various sarcoma cell lines treated with serial dilutions of VasG3 antibody compared to control isotype antibody. *A*. aRMS cell lines: Rh30, PCB380, and Rh5. *B*. Cell viability assays of Rh30 aRMS cells treated with a therapeutic combination of VasG3 and the pan-kinase inhibitor, midostaurin. *C*. Osteosarcoma cell lines: PCB151 and U-2 OS. *D*. Neuroblastoma cell line: SH-SY5Y. All assays were performed in quadruplicate.
(TIF)

S2 Fig Expression of EphB4 and EphrinB2 in osteosarcoma and neuroblastoma. *A*, *B*. Immunohistochemical staining for EphB4 and EphrinB2 expression was performed on tissue microarrays for human neuroblastoma (A) and osteosarcoma (B). Staining intensities are shown for EphB4 and EphrinB2 on a scale ranging from 0 (no staining) to 4 (high staining). *C*, *D*. Representative immunohistochemistry staining of 4^+^ EphB4 and EphrinB2 in human neuroblastoma (C) and osteosarcoma (D) biopsies. Mag bar = 100 μm. *E*. Western blots of several human primary neuroblastoma and osteosarcoma tumors. EphB4 and EphrinB2 proteins were both present in most of the tested samples.
(TIF)

## References

[1] Arndt CA, Crist WM. Common musculoskeletal tumors of childhood and adolescence. N Engl J Med. 1999;341(5):342–52. doi: 10.1056/NEJM199907293410507 . 10423470 10.1056/NEJM199907293410507

[2] Rodeberg DA, Garcia-Henriquez N, Lyden ER, Davicioni E, Parham DM, Skapek SX, et al Prognostic significance and tumor biology of regional lymph node disease in patients with rhabdomyosarcoma: a report from the Children's Oncology Group. J Clin Oncol. 2011;29(10):1304–11. doi: 10.1200/JCO.2010.29.4611 ; PubMed Central PMCID: PMCPMC3083998. 21357792 10.1200/JCO.2010.29.4611 PMC3083998

[3] Davicioni E, Anderson JR, Buckley JD, Meyer WH, Triche TJ. Gene expression profiling for survival prediction in pediatric rhabdomyosarcomas: a report from the children's oncology group. J Clin Oncol. 2010;28(7):1240–6. doi: 10.1200/JCO.2008.21.1268 ; PubMed Central PMCID: PMCPMC3040045. 20124188 10.1200/JCO.2008.21.1268 PMC3040045

[4] Zwick E, Bange J, Ullrich A. Receptor tyrosine kinase signalling as a target for cancer intervention strategies. Endocr Relat Cancer. 2001;8(3):161–73. . 11566607 10.1677/erc.0.0080161

[5] Pasquale EB. Eph receptors and ephrins in cancer: bidirectional signalling and beyond. Nat Rev Cancer. 2010;10(3):165–80. doi: 10.1038/nrc2806 ; PubMed Central PMCID: PMCPMC2921274. 20179713 10.1038/nrc2806 PMC2921274

[6] Berardi AC, Marsilio S, Rofani C, Salvucci O, Altavista P, Perla FM, et al Up-regulation of EphB and ephrin-B expression in rhabdomyosarcoma. Anticancer Res. 2008;28(2A):763–9. . 18507018

[7] Bai Y, Li J, Fang B, Edwards A, Zhang G, Bui M, et al Phosphoproteomics identifies driver tyrosine kinases in sarcoma cell lines and tumors. Cancer Res. 2012;72(10):2501–11. doi: 10.1158/0008-5472.CAN-11-3015 ; PubMed Central PMCID: PMCPMC4641440. 22461510 10.1158/0008-5472.CAN-11-3015 PMC4641440

[8] Salvucci O, de la Luz Sierra M, Martina JA, McCormick PJ, Tosato G. EphB2 and EphB4 receptors forward signaling promotes SDF-1-induced endothelial cell chemotaxis and branching remodeling. Blood. 2006;108(9):2914–22. doi: 10.1182/blood-2006-05-023341 ; PubMed Central PMCID: PMCPMC1895526. 16840724 10.1182/blood-2006-05-023341 PMC1895526

[9] Aslam MI, Abraham J, Mansoor A, Druker BJ, Tyner JW, Keller C. PDGFRbeta reverses EphB4 signaling in alveolar rhabdomyosarcoma. Proc Natl Acad Sci U S A. 2014;111(17):6383–8. doi: 10.1073/pnas.1403608111 ; PubMed Central PMCID: PMC4035936. 24733895 10.1073/pnas.1403608111 PMC4035936

[10] Krasnoperov V, Kumar SR, Ley E, Li X, Scehnet J, Liu R, et al Novel EphB4 monoclonal antibodies modulate angiogenesis and inhibit tumor growth. Am J Pathol. 2010;176(4):2029–38. doi: 10.2353/ajpath.2010.090755 ; PubMed Central PMCID: PMCPMC2843490. 20133814 10.2353/ajpath.2010.090755 PMC2843490

[11] Hooper JE, Cantor EL, Ehlen MS, Banerjee A, Malempati S, Stenzel P, et al A Patient-Derived Xenograft Model of Parameningeal Embryonal Rhabdomyosarcoma for Preclinical Studies. Sarcoma. 2015;2015:826124 doi: 10.1155/2015/826124 ; PubMed Central PMCID: PMCPMC4677247. 26696773 10.1155/2015/826124 PMC4677247

[12] Davicioni E, Finckenstein FG, Shahbazian V, Buckley JD, Triche TJ, Anderson MJ. Identification of a PAX-FKHR gene expression signature that defines molecular classes and determines the prognosis of alveolar rhabdomyosarcomas. Cancer Res. 2006;66(14):6936–46. doi: 10.1158/0008-5472.CAN-05-4578 . 16849537 10.1158/0008-5472.CAN-05-4578

[13] Sokolowski E, Turina CB, Kikuchi K, Langenau DM, Keller C. Proof-of-concept rare cancers in drug development: the case for rhabdomyosarcoma. Oncogene. 2014;33(15):1877–89. doi: 10.1038/onc.2013.129 . 23665679 10.1038/onc.2013.129

[14] Rodriguez-Perales S, Martinez-Ramirez A, de Andres SA, Valle L, Urioste M, Benitez J, et al Molecular cytogenetic characterization of rhabdomyosarcoma cell lines. Cancer Genet Cytogenet. 2004;148(1):35–43. . 14697639 10.1016/s0165-4608(03)00216-4

[15] Schneider CA, Rasband WS, Eliceiri KW. NIH Image to ImageJ: 25 years of image analysis. Nat Methods. 2012;9(7):671–5. . 22930834 10.1038/nmeth.2089 PMC5554542

[16] Kertesz N, Krasnoperov V, Reddy R, Leshanski L, Kumar SR, Zozulya S, et al The soluble extracellular domain of EphB4 (sEphB4) antagonizes EphB4-EphrinB2 interaction, modulates angiogenesis, and inhibits tumor growth. Blood. 2006;107(6):2330–8. doi: 10.1182/blood-2005-04-1655 ; PubMed Central PMCID: PMCPMC1895726. 16322467 10.1182/blood-2005-04-1655 PMC1895726

[17] Thomas SM, Brugge JS. Cellular functions regulated by Src family kinases. Annu Rev Cell Dev Biol. 1997;13:513–609. doi: 10.1146/annurev.cellbio.13.1.513 . 9442882 10.1146/annurev.cellbio.13.1.513

[18] Nada S, Yagi T, Takeda H, Tokunaga T, Nakagawa H, Ikawa Y, et al Constitutive activation of Src family kinases in mouse embryos that lack Csk. Cell. 1993;73(6):1125–35. . 8513497 10.1016/0092-8674(93)90642-4

[19] Georgakopoulos A, Litterst C, Ghersi E, Baki L, Xu C, Serban G, et al Metalloproteinase/Presenilin1 processing of ephrinB regulates EphB-induced Src phosphorylation and signaling. EMBO J. 2006;25(6):1242–52. doi: 10.1038/sj.emboj.7601031 ; PubMed Central PMCID: PMCPMC1422162. 16511561 10.1038/sj.emboj.7601031 PMC1422162

[20] Blandford MC, Barr FG, Lynch JC, Randall RL, Qualman SJ, Keller C. Rhabdomyosarcomas utilize developmental, myogenic growth factors for disease advantage: a report from the Children's Oncology Group. Pediatr Blood Cancer. 2006;46(3):329–38. doi: 10.1002/pbc.20466 . 16261596 10.1002/pbc.20466

[21] Xia G, Kumar SR, Stein JP, Singh J, Krasnoperov V, Zhu S, et al EphB4 receptor tyrosine kinase is expressed in bladder cancer and provides signals for cell survival. Oncogene. 2006;25(5):769–80. doi: 10.1038/sj.onc.1209108 . 16205642 10.1038/sj.onc.1209108

[22] Oricchio E, Nanjangud G, Wolfe AL, Schatz JH, Mavrakis KJ, Jiang M, et al The Eph-receptor A7 is a soluble tumor suppressor for follicular lymphoma. Cell. 2011;147(3):554–64. doi: 10.1016/j.cell.2011.09.035 ; PubMed Central PMCID: PMCPMC3208379. 22036564 10.1016/j.cell.2011.09.035 PMC3208379

[23] Noren NK, Foos G, Hauser CA, Pasquale EB. The EphB4 receptor suppresses breast cancer cell tumorigenicity through an Abl-Crk pathway. Nat Cell Biol. 2006;8(8):815–25. doi: 10.1038/ncb1438 . 16862147 10.1038/ncb1438

[24] Batlle E, Bacani J, Begthel H, Jonkheer S, Gregorieff A, van de Born M, et al EphB receptor activity suppresses colorectal cancer progression. Nature. 2005;435(7045):1126–30. doi: 10.1038/nature03626 . 15973414 10.1038/nature03626

[25] Masood R, Kumar SR, Sinha UK, Crowe DL, Krasnoperov V, Reddy RK, et al EphB4 provides survival advantage to squamous cell carcinoma of the head and neck. Int J Cancer. 2006;119(6):1236–48. doi: 10.1002/ijc.21926 . 16615113 10.1002/ijc.21926

[26] Noren NK, Lu M, Freeman AL, Koolpe M, Pasquale EB. Interplay between EphB4 on tumor cells and vascular ephrin-B2 regulates tumor growth. Proc Natl Acad Sci U S A. 2004;101(15):5583–8. doi: 10.1073/pnas.0401381101 ; PubMed Central PMCID: PMCPMC397426. 15067119 10.1073/pnas.0401381101 PMC397426

