# Supplementary figures and images for: EphB4/EphrinB2 therapeutics in Rhabdomyosarcoma
Source: PLoS One. 2017 Aug 17;12(8):e0183161. doi: 10.1371/journal.pone.0183161 (PMC5560593; doi:10.1371/journal.pone.0183161)

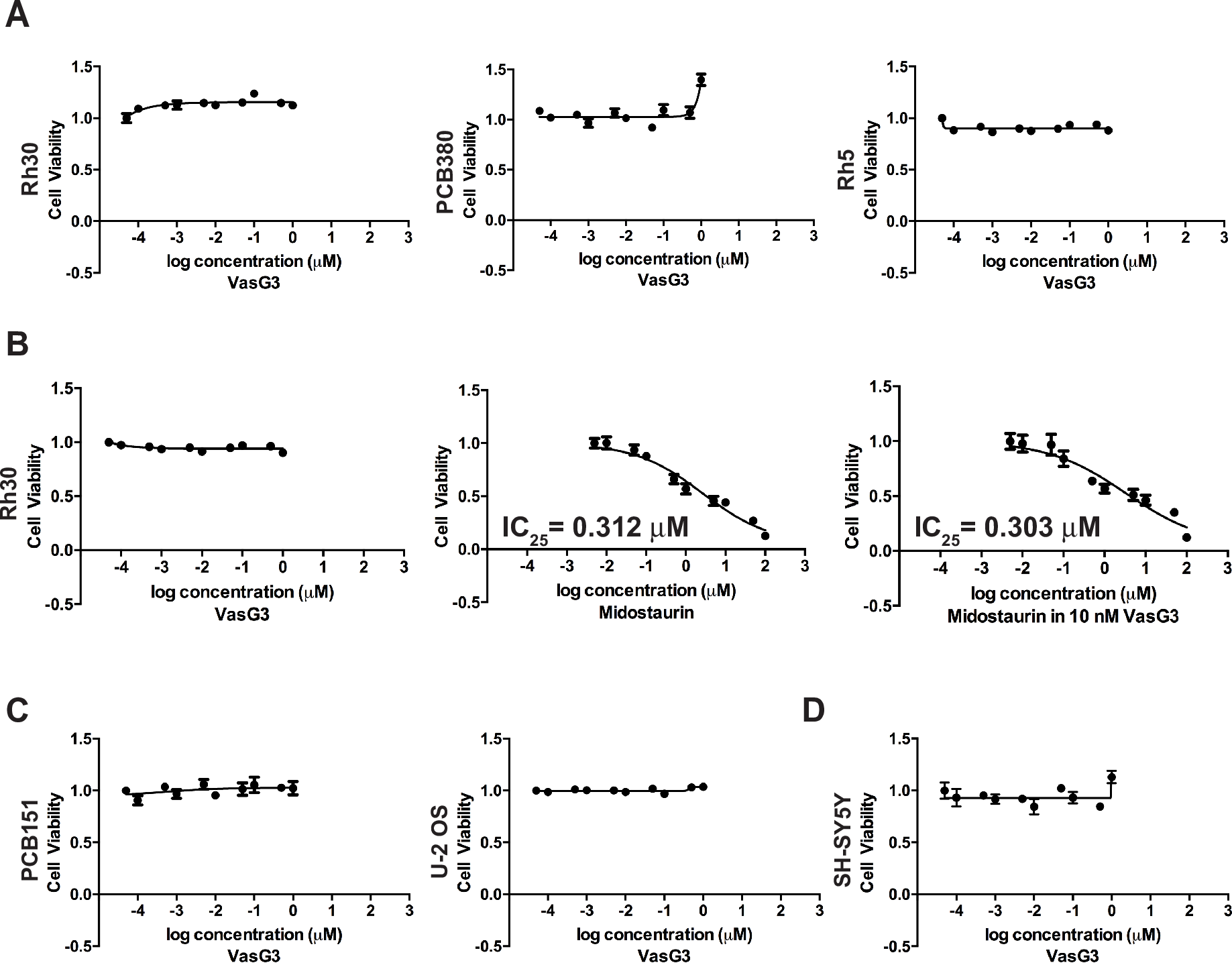

Supplement: S1 Fig — Cell viability of various sarcoma cell lines treated with serial dilutions of VasG3 antibody compared to control isotype antibody. A. aRMS cell lines: Rh30, PCB380, and Rh5. B. Cell viability assays of Rh30 aRMS cells treated with a therapeutic combination of VasG3 and the pan-kinase inhibitor, midostaurin. C. Osteosarcoma cell lines: PCB151 and U-2 OS. D. Neuroblastoma cell line: SH-SY5Y. All assays were performed in quadruplicate. (TIF) [file pone.0183161.s001.tif]

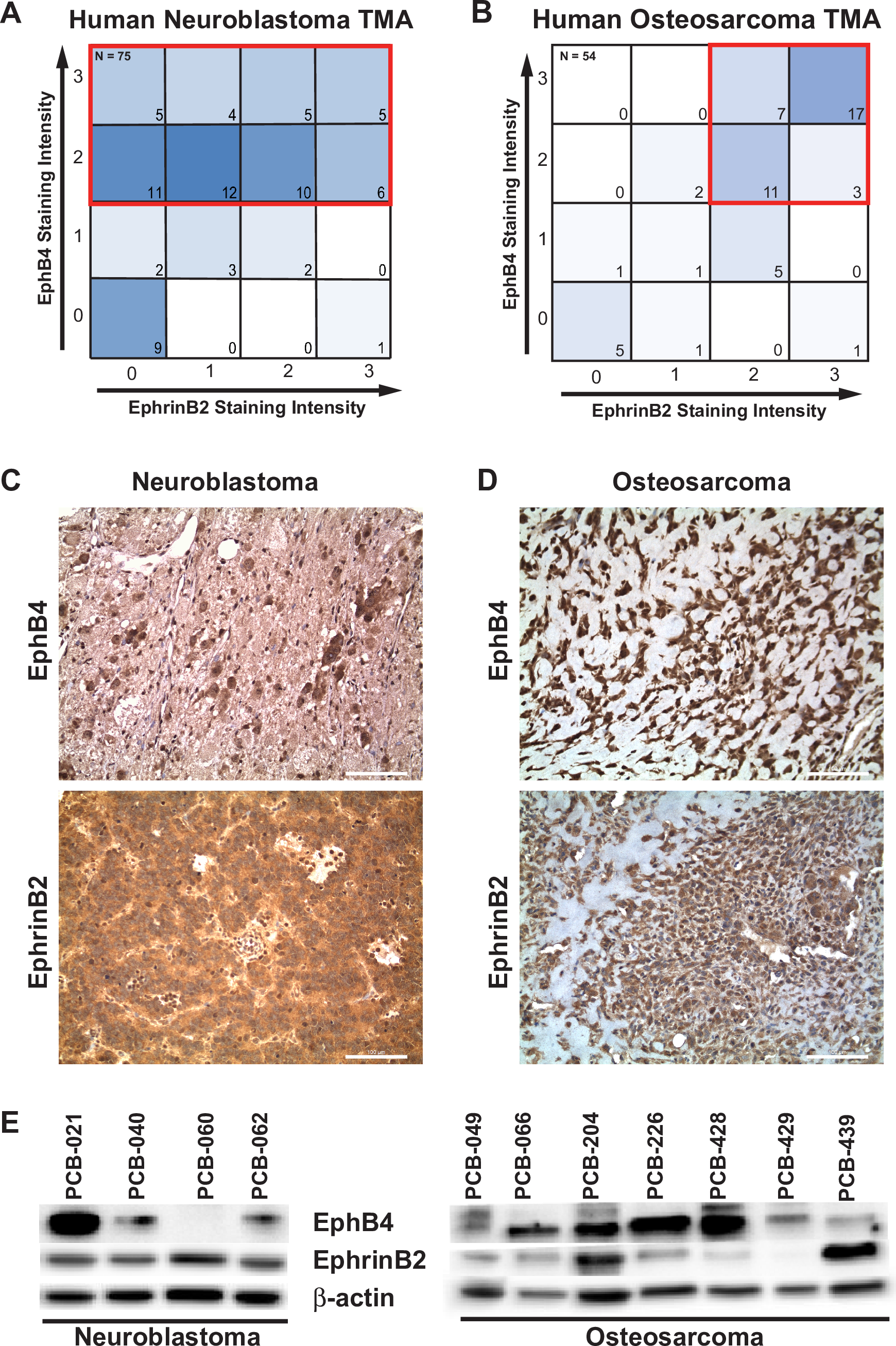

Supplement: S2 Fig — A, B. Immunohistochemical staining for EphB4 and EphrinB2 expression was performed on tissue microarrays for human neuroblastoma (A) and osteosarcoma (B). Staining intensities are shown for EphB4 and EphrinB2 on a scale ranging from 0 (no staining) to 4 (high staining). C, D. Representative immunohistochemistry staining of 4+ EphB4 and EphrinB2 in human neuroblastoma (C) and osteosarcoma (D) biopsies. Mag bar = 100 μm. E. Western blots of several human primary neuroblastoma and osteosarcoma tumors. EphB4 and EphrinB2 proteins were both present in most of the tested samples. (TIF) [file pone.0183161.s002.tif]
